# Comparison of Risk Factors and Disease Severity Between Old and Young Patients With Gastroesophageal Reflux Disease

**DOI:** 10.4021/gr549w

**Published:** 2013-07-14

**Authors:** Shou-Wu Lee, Teng-Yu Lee, Han-Chung Lien, Hong-Zen Yeh, Chi-Sen Chang, Chung-Wang Ko

**Affiliations:** a Division of Gastroenterology, Department of Internal Medicine, Taichung Veterans General Hospital, Taichung, Taiwan; b Department of Internal Medicine, Chung Shan Medical University, Taichung, Taiwan; c Department of Internal Medicine, Yang-Ming University, Taipei, Taiwan

**Keywords:** Elderly, Gastroesophageal reflux disease, Risk factor

## Abstract

**Background:**

Gastroesophageal reflux disease (GERD) tends to relapse and develop complications. The aim of the study was to compare the risk factors and disease severity of GERD in young and old patients.

**Methods:**

Data from patients with GERD were collected between January and November 2009. The enrolled cases were assigned to the younger group if they were below 65 years, or the elderly group if 65 years or older. The general demographic data, lifestyle characteristics and endoscopic findings of the two groups were compared.

**Results:**

Among all enrolled 111 patients, 78 and 33 patients were classified in the younger and elderly groups, respectively. The elderly group had significantly more men than the younger group did (72.7% vs 39.7%, P = 0.001). Lower rates of smoking (3% vs 6.4%, P = 0.029) and tea drinking (21.3% vs 34.6%, P = 0.001) were noted in the elderly patients, but similar rates of alcohol and coffee drinking. There were more severe esophagitis, esophagocardiac junction (ECJ) ulcers (21.2% vs. 2.6%, P = 0.003) and hiatal hernia (36.4% vs 16.9%, P = 0.025) in the elderly group.

**Conclusion:**

Elderly GERD patients were more likely to be male, and having severe esophagitis, but lower rates of cigarette smoking and tea drinking, than those of younger patients.

## Introduction

Gastroesophageal reflux disease (GERD), defined by the Montreal criteria as ‘a condition which develops when the reflux of stomach contents causes troublesome symptoms or complications’ [1], is a chronic disease that tends to relapse and develop complications. It is reported to be more severe and to have a higher incidence of severe complications in old versus young or adult patients [2-4]. The prevalence of the disease increases with age [3, 5], and advanced age has been identified as a significant risk factor for relapse of esophagitis [4-7]. According to previous studies, the risk factors in patients with GERD included pregnancy, obesity, hiatal hernia, unfavorable liefestyle, such as alcohol drinking and cigarette smoking [8-13]. However, at present, no studies have been performed to assess risk factors between young and old patients with GERD. The aim of the study was to compare the risk factors and endoscopic severity of GERD between elderly and younger patients.

## Methods

Data from consecutive patients with GERD, diagnosed by Montreal definition, in our hospital were collected from January 2009 to November 2009. All patients underwent an open-access transoral upper gastrointestinal endoscopy, and those who were diagnosed with erosive esophagitis or had typical symptoms were considered for inclusion in the study. Exclusion criteria were as follows: (1) GERD combined with other structural gastrointestinal disorders, such as peptic ulcer disease, esophageal or gastric malignancy; (2) prior gastric surgery; (3) use of chronic anti-acid medication, such as proton pump inhibitors (PPIs) or H2-receptor antagonists (H2RAs), for more than 2 months prior to enrollment, and (4) pregnancy.

The enrolled patients were assigned to the younger group (under 65 years) or the elderly group (65 years or older). The general data, lifestyle characteristics and endoscopic findings of the two groups were recorded and compared.

Data are expressed as standard derivation of mean for each of the measured parameters. Gender is expressed as a percentage of the total patient number. A P value below 0.05 was considered statistically significant. Statistical comparisons were made using Pearson’s chi-square test to compare the effects of gender, lifestyle items and endoscopic findings characteristic; Independent T test was used to analyze scores of body weight and BMI (body mass index).

## Results

A total of 111 consecutive patients were enrolled between January and November 2009, with 78 (70.3%) and 33 patients (29.7%) in the younger group and the elderly group, respectively. Comparing the gender of each group, as displayed in Table 1, there were significantly more male patients (72.7%) in the elderly group, and more female patients (60.3%) in the younger group. Similar values of body weight and BMI were noted between the elderly patients (mean 64.32 kg, 23.43 kg/m^2^) and the younger patients (mean 63.88 kg, 23.35 kg/m^2^).

**Table 1:** Basic Characteristic of the Elderly and Younger Patients With GERD

Variable	Elderly group (n = 33)			Younger group (n = 78)			P-value
	n	%	M ± SD	n	%	M ± SD	
Mean age (year)			72.94 ± 9.58			37.73 ± 10.17	
Gender							0.001^a^
M	24	72.7%		31	39.7%		
F	9	27.3%		47	60.3%		
Weight (kg)			63.88 ± 13.29			64.32 ± 13.35	0.874^b^
BMI (kg/m^2^)			23.35 ± 3.90			23.43 ± 3.98	0.917^b^
Coffee	15	45.4%		36	46.2%		0.292^a^
Alcohol	13	39.4%		31	39.7%		0.083^a^
Tea	7	21.3%		27	34.6%		0.001^a^
Smoke	1	3.0%		5	6.4%		0.029^c^

^a^Pearson’s Chi-square test; ^b^T test; ^c^Fisher’s exact test. BMI: nody mass index; F: female; M: male; M ± SD: mean ± standard deviation; n: numbers.

The lifestyle characteristics between the younger and elderly patients with GERD in our study are also shown in Table 1. Significantly lower rates of cigarette smoking (3% vs 6.4%, P = 0.029) and tea drinking (21.3% vs 34.6%, P = 0.001) were noted in the elderly patients compared with those in the younger group, but there were similar rates of alcohol (39.4% vs 39.7%, P = 0.083) and coffee drinking (45.4% vs 46.2%, P = 0.292) between the two groups.

The endoscopic findings between the two groups are summarized Table 2. The rates of *H. pylori* infection were non-significantly different between the elderly and younger patients with GERD (30.3% vs 24.3%, P = 0.515). However, there was a significantly higher rate of hiatal hernia in the elderly group (36.4% vs 16.9%, P = 0.025). Furthermore, the elderly patients had greater disease severity than the younger patients based on Los Angeles classification (L.A. grade C/D, 27.3% vs. 6.4%, P = 0.001), and presence of esophagocardiac junction (ECJ) ulcers (21.2% vs. 2.6%, P = 0.003).

**Table 2:** Endosocpic Severity of the Elderly and Younger Patients With GERD

Variable	Elderly group (n = 33)		Younger group (n = 78)		P-value
	n	%	n	%	
H. pylori infection	10	30.3%	19	24.4%	0.515^a^
Hiatal hernia	12	36.4%	13	16.9%	0.025^a^
ECJ ulcer	7	21.2%	2	2.6%	0.003^b^
L.A. grade					0.001^b^
NERD	0		15	19.2%	
A/B	24	82.7%	58	74.4%	
C/D	9	27.3%	5	6.4%	

^a^Pearson’s Chi-square test; ^b^Fisher’s exact test. ECJ: esophaocardiac junction; L.A. grade: Los Angeles grade; n: numbers; NERD: non-erosive reflux disease.

## Discussion

GERD is a chronic disease which has an impact on the everyday lives of affected individuals, and old age was found to be a significant risk factor in the development of severe forms of GERD in epidemiological and clinical studies in the United States, Japan, and Europe [8, 14-16]. In addition, previous studies demonstrated that a higher incidence of and more severe complications existed in old patients with GERD [2, 3]. Our study investigated risk factors and severity of disease in elderly and younger patients with GERD.

According to previous epidemiological reports, male gender is not the sole risk factor in GERD [8, 11, 13], as obesity [8, 10, 11, 13, 17], hiatal hernia [18], and unfavorable lifestyle [8, 10, 11], including alcohol drinking and cigarette smoking, have also been shown to increase risk of developing the disease. However, infection with *H. pylori* is thought to play a protective role in GERD, perhaps due to the corresponding occurrence of atrophic gastritis [19, 20].

Our study’s findings showed that most old patients with GERD had a similar body weight, BMI and rate of *H. pylori* infection compared with younger patients, but had a significantly higher rate of hiatal hernia, and a lower incidence of cigarette smoking. Furthermore, elderly patients with GERD were predominantly male. Hence, the more meaningful risk factors of GERD in the elderly are male gender and hiatal hernia, but obesity and unfavorable lifestyle did not appear to be risk factors. It is important to recognize that GERD in older patients may have a different pathophysiology from that in younger patients.

As shown in previous reports [2-4, 13, 15, 17, 21], our results demonstrated more advanced endoscopic severity of GERD in elderly patients compared with younger patients, which was estimated not only by Los Angeles classification but also by ECJ ulcers ratios. The reason for these differences may be due to most typical symptoms of GERD occurring in the young patients, but being rarer in older patients [21]. Hence, the older patients with GERD were identified and diagnosed in the late stage of disease. Our study findings indicate that early diagnosis of GERD is especially desirable in elderly patients, therefore prompt evaluation is necessary should any signs or symptoms present themselves.

There were some limitations in our study. Firstly, the lifestyle characteristics in our study were only limited to the patients’ current status, and the past history of each case was not taken. Secondly, co-morbidity diseases of these patients that tend to influence severity of GERD, such as chronic heart failure or chronic obstructive pulmonary disease, were not considered, and this might have led to inaccurate outcomes. Lastly, our study’s design was hospital-based. Further research in representative samples of the general population are needed to confirm these results.

### Conclusion

In the present study, elderly patients with GERD were prodominantly male, and had higher incidence rates of severe esophagitis, ECJ ulcers and hiatal hernia than those in the younger group, but there were lower rates of cigarette smoking and tea drinking in the older group.
